# The portal‐drained viscera release fibroblast growth factor 19 in humans

**DOI:** 10.14814/phy2.13037

**Published:** 2016-12-21

**Authors:** Kiran V. K. Koelfat, Johanne G. Bloemen, Peter L. M. Jansen, Cornelis H. C. Dejong, Frank G. Schaap, Steven W. M. Olde Damink

**Affiliations:** ^1^ Department of SurgeryMaastricht University Medical Center and NUTRIM School of Nutrition and Translational Research in MetabolismMaastricht UniversityMaastrichtThe Netherlands; ^2^ GROW School for Oncology and Developmental BiologyMaastricht UniversityMaastrichtThe Netherlands; ^3^ Department of HPB Surgery & Liver TransplantationInstitute for Liver and Digestive HealthUniversity College LondonLondonUnited Kingdom

**Keywords:** bile salts, entero‐hepatic circulation, FGF19, organ fluxes

## Abstract

Fibroblast growth factor 19 (FGF19) is an ileum‐derived endrocrine factor that is produced in response to transepithelial bile salt flux. FGF19 represses bile salt synthesis in the liver. Despite the general assumption that FGF19 signals to the liver via portal blood, no human data are available to support this notion. The aim was to study portal FGF19 levels, and determined bile salt and FGF19 fluxes across visceral organs in humans. Bile salt and FGF19 levels were assessed in arterial, portal, and hepatic venous blood collected from fasted patients who underwent partial liver resection for colorectal liver metastases (*n* = 30). Fluxes across the portal‐drained viscera (PDV), liver, and splanchnic area were calculated. Portal bile salt levels (7.8 [5.0–12.4] *μ*mol/L) were higher than levels in arterial (2.7 [1.7–5.5] *μ*mol/L, *P *<* *0.0001) and hepatic venous blood (3.4 [2.5–6.5] *μ*mol/L, *P *<* *0.0001). Bile salts released by the PDV (+1.2 [+0.7–+2.0] mmol kg^−1^ h^−1^, *P *<* *0.0001) were largely taken up by the liver (−1.0 [−1.8 to −0.4] mmol kg^−1^ h^−1^, *P *<* *0.0001). Portal levels of FGF19 (161 ± 78 pg/mL) were higher than arterial levels (135 ± 65 pg/mL, *P *=* *0.046). A net release of FGF19 by the PDV (+4.0 [+2.1 to +9.9] ng kg^−1^ h^−1^, *P *<* *0.0001) was calculated. There was no significant flux of FGF19 across the liver (−0.2 [−3.7 to +7.4] ng kg^−1^ h^−1^, *P *=* *0.93). In conclusion, FGF19 levels in human portal blood are higher than in arterial blood. FGF19 is released by the portal‐drained viscera under fasted steady state conditions.

## Introduction

Related to their role in digestion and absorption of dietary lipids, bile salts are signaling molecules engaged in regulation of postprandial nutrient handling (Kuipers et al. 2014). Bile salt signaling is mediated by dedicated receptors expressed at the plasma membrane or inside the cell (e.g., Farnesoid X Receptor, FXR) (Schaap et al. 2014). FXR is a bile salt‐activated transcription factor that is abundantly expressed in the terminal ileum (i.e., enterocytes) and the liver (i.e., hepatocytes) (Inagaki et al. 2006; Kim et al. 2007). Besides (postprandial) control of hepatic metabolism, bile salt/FXR signaling is implicated in other processes including gut barrier integrity and liver regeneration (Gadaleta et al. 2011; Zhang et al. 2012).

Bile salt levels in the liver are tightly controlled to prevent bile salt toxicity. FXR plays a central role in this process by mediating negative feedback inhibition of bile salt synthesis (Schaap et al. 2014). Regulation of bile salt synthesis occurs primarily at the transcriptional level by controlling expression of the rate‐limiting enzyme in the biosynthetic pathway, viz. *CYP7A1* (Goodwin et al. 2000; Lu et al. 2000). Thus, bile salts released after a meal activate FXR in the ileum and this results in the induction of *FGF19/15* (Fibroblast Growth Factor 19/15, *Fgf15* is the rodent ortholog of human FGF19). Subsequent binding of FGF19/15 to FGFR4 in the liver results in activation of intracellular signaling cascades that target expression of *CYP7A1,* thereby lowering bile salt synthesis (Song et al. 2009).

Direct activation of hepatic FXR with concomitant induction of a transcriptional repressor that downregulates *CYP7A1* may be an alternative route to control bile salt synthesis (Goodwin et al. 2000). However, studies in mice with tissue‐specific disruption of *Fxr* indicate that the ileal Fxr/Fgf15 pathway is the primary regulator of bile salt synthesis in the fed state (Kong et al. 2012; Inagaki et al. 2005). This implies that portal levels of Fgf15 should increase postprandially. Technical challenges to detect and quantify Fgf15 protein were overcome only recently, and it was demonstrated that portal Fgf15 levels indeed increased after oral administration of an Fxr agonist (Katafuchi et al. 2015). While postprandial elevation of FGF19 in the systemic circulation is readily noted in humans (Schreuder et al. 2010), no data are available on portal FGF19 levels thus far. Moreover, FGF19 has been reported to follow a diurnal rhythm which could be largely explained by food intake (Lundasen et al. 2006). Thus far, portal levels were neither studied in the fed or fasted state. In this study we aimed to provide evidence for FGF19 release by the human portal‐drained viscera under fasted conditions.

## Methods

### Patient materials

Plasma samples from patients undergoing liver surgery for colorectal liver metastasis (*n* = 30), were selected from a larger cohort of patients that underwent liver surgery at our center between October 2007 and June 2009. The larger cohort and the per‐operative blood sampling procedure have been described previously (Bloemen et al. 2009). In brief, patients were prepared for abdominal surgery according to institutional procedures. Patients were fasted for 12–16 h prior to surgery. Within 1 h after laparotomy, blood was near‐simultaneously drawn from the portal vein [PV], middle hepatic vein [HV], and an arterial line [A]. Blood was collected in EDTA tubes, and plasma samples were biobanked at −80°C. The study was approved by the Medical Ethical Committee of Maastricht University Medical Center. All patients gave written informed consent.

### Analytical procedures

FGF19 was assayed by sandwich ELISA as described previously (Schaap et al. 2009). Total bile salts were determined using an enzymatic cycling method according to the manufacturer's protocol (Diazyme Laboratories, Poway, CA, USA).

### Calculations

Fluxes were calculated across the portal‐drained viscera (PDV, comprising the intestines, stomach, spleen, and pancreas), the liver, and the splanchic area (Bloemen et al. 2009; van de Poll et al. 2007). In brief, venous‐arterial differences (ΔVA) were calculated for the portal‐drained viscera (ΔVA_PDV_ = [PV]–[A]) and splanchnic area (ΔVA_SPL_ = [HV]–[A]). To calculate fluxes*,* ΔVA was multiplied with the corresponding plasma flow rate. Assumed plasma flow rates for the portal vein (320 mL/min), hepatic artery (110 mL/min), and splanchnic area (portal vein flow rate + hepatic artery flow rate = 430 mL/min) were based on historical measurements of the respective blood flow rates by Doppler ultrasound in an unrelated cohort of surgical patients (van de Poll et al. 2007). Liver flux was derived from the relation between fluxes across the splanchnic area and the PDV (liver flux = splanchnic flux–PDV flux). Fluxes were corrected for body weight. A positive flux indicates net release and a negative flux indicates net uptake.

### Statistics

Differences in levels of bile salts and FGF19 in the three blood vessels were evaluated with one‐way ANOVA with Tukey's multiple comparison test assuming normal distribution of the data, or a Friedman test with Dunn's multiple comparison test in case of nonparametric distribution. The median flux was tested against a theoretical value of zero using the Wilcoxon signed rank test. Correlations were evaluated by Pearson (r) or Spearman (*ρ*) correlation coefficient. Data are expressed as mean ± standard deviation or median [interquartile range], and are presented as box and whisker plots with 10th and 90th percentile unless indicated otherwise. *P* values below 0.05 were considered statistically significant. Statistical analyses were performed using GraphPad Prism 6.0 (GraphPad Software Inc., San Diego, CA, USA) and SPSS 22.0 (IBM SPSS Inc, Chicago, IL, USA).

## Results

### Patient characteristics

Plasma samples from 30 patients who underwent liver resection for colorectal metastases were analyzed in this study (Table 1). Patients displayed normal liver tests and were noncholestatic. Twelve patients had higher preoperative gamma glutamyl transferase (*γ*GT) levels than the upper limit of the normal range (5–50 IU/L) (Table 1). All patients were fasted overnight according to institutional guidelines.

**Table 1:** Patient characteristics

Patients (*n*)	30
Male/female (*n*)	15/15
Age (years)	63 ± 12
BMI (kg/m^2^)	25.6 ± 6.7
Preoperative blood values:	
ALT (IU/L)	26.8 ± 11.8
AST (IU/L)	31.1 ± 14.8
Bilirubin (*μ*mol/L)	13.7 ± 5.5
*γ*GT (IU/L)	65.8 ± 55.8

Data are expressed as mean ± standard deviation; BMI, body mass index; ALT, alanine aminotransferase; AST, aspartate aminotransferase; *γ*GT, gamma glutamyl transferase.

### Bile salt and FGF19 levels in portal, arterial, and hepatic venous blood

**Figure 1:**
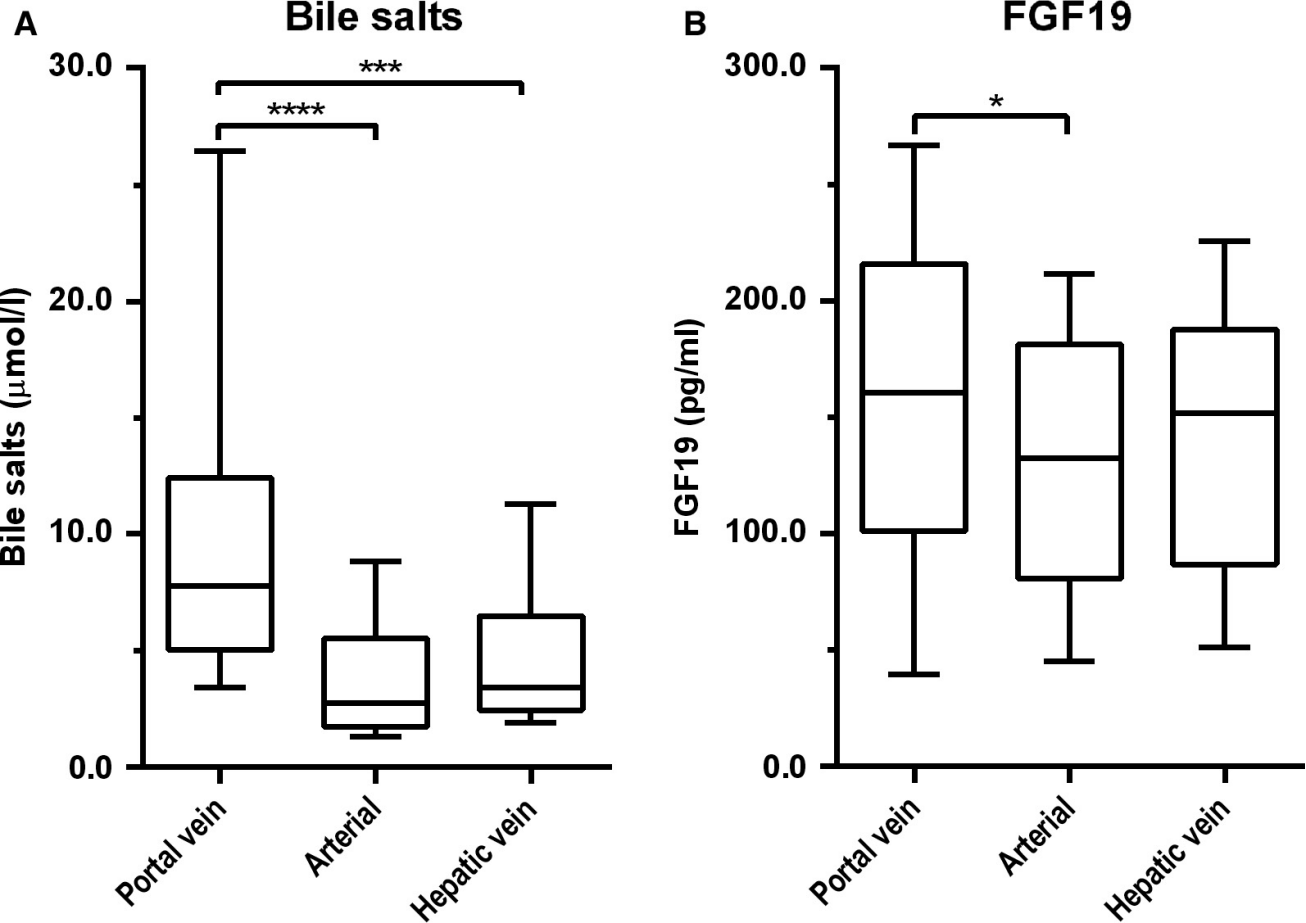
Blood was drawn concurrently from three vessels during abdominal surgery of patients with colorectal liver metastases (*n* = 30). Plasma was assayed for total bile salts (A) and FGF19 (B). Data are presented as box and whisker plots with 10th and 90th percentile. *(*P *<* *0.05), *** (*P *<* *0.001), and ****(*P *<* *0.0001).

**Figure 2:**
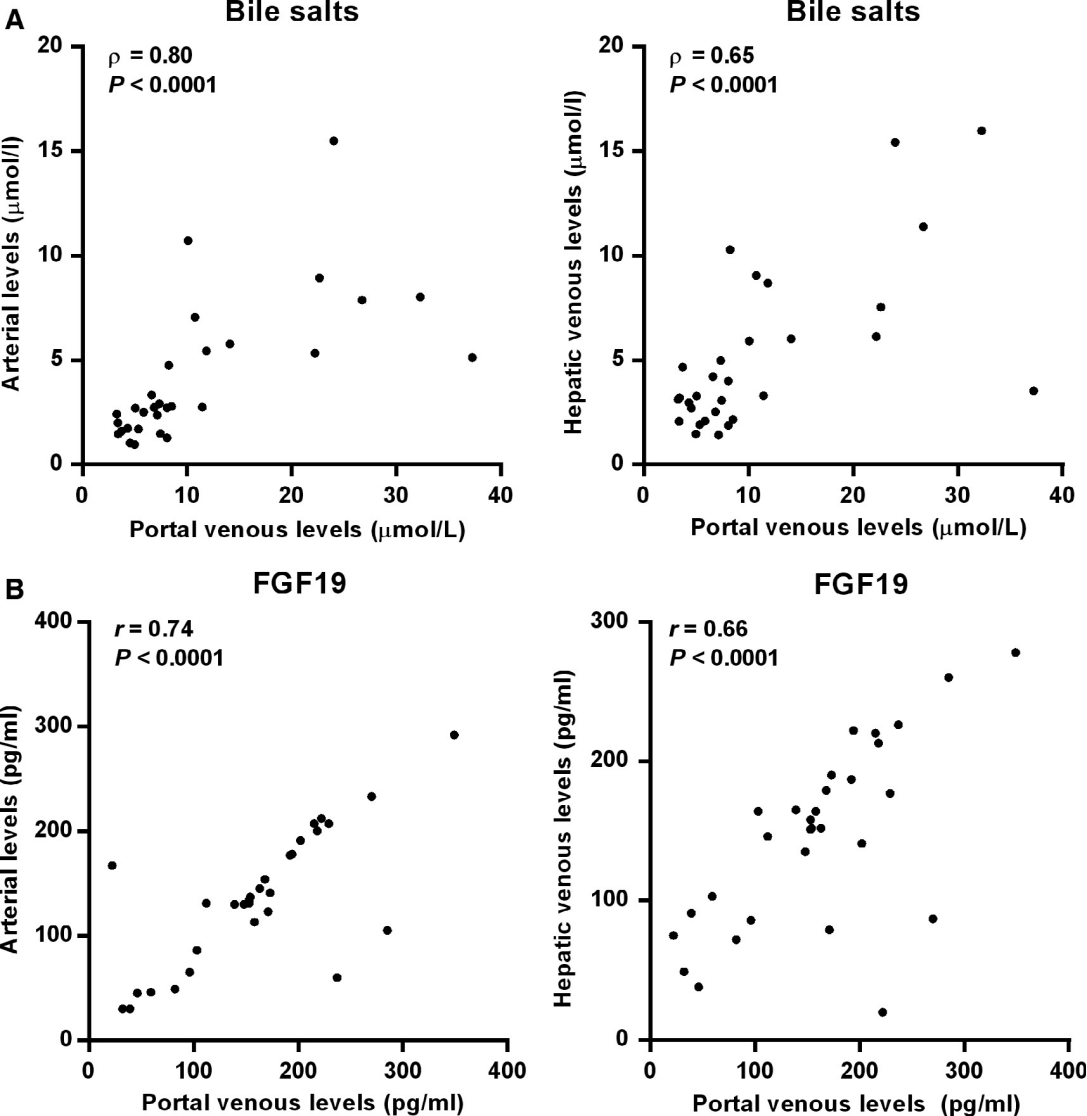
Relationship between portal levels and arterial and hepatic venous levels of (A) bile salts and (B) FGF19. Pearson or Spearman correlation coefficients and *P* values are shown.

**Figure 3:**
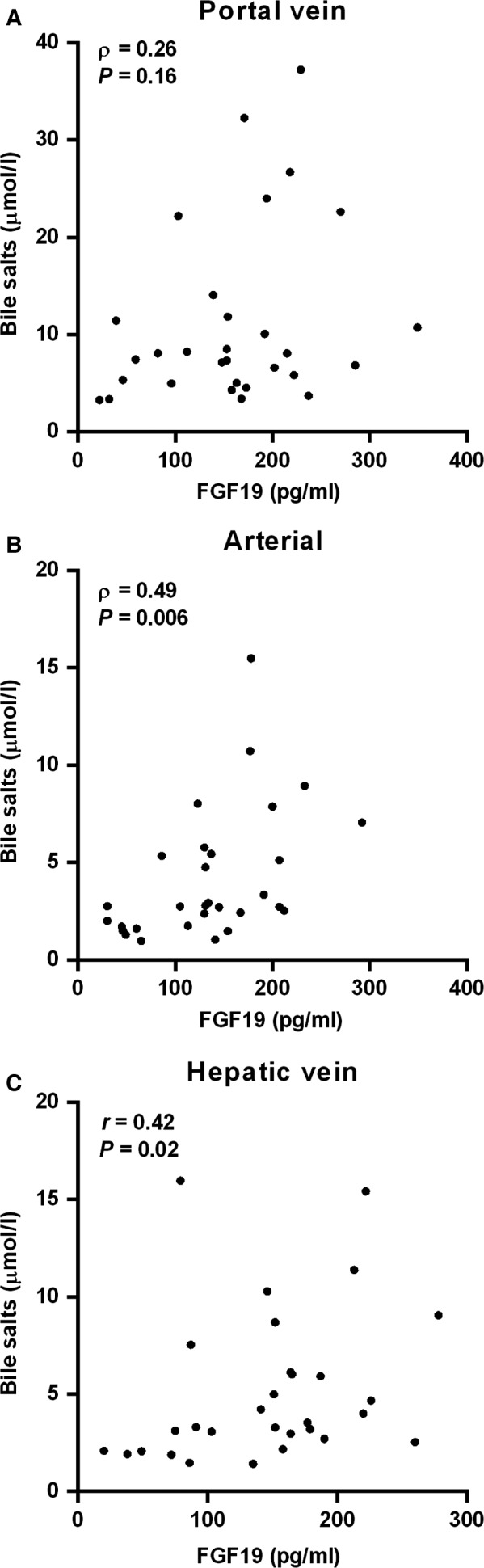
Relationship between bile salts and FGF19 levels in (A) the portal vein, (B) arterial blood, and (C) hepatic vein. Spearman correlation coefficients and *P* values are shown.

Bile salts reclaimed from the intestinal lumen are secreted into the portal tributaries and extracted by the liver for resecretion into bile. Enterohepatic recirculation effectively conserves the bile salt pool, with minor losses via feces compensated for by de novo synthesis (Hofmann and Hagey 2014). Under fasted conditions, bile salt levels were higher in the portal vein (7.8 [5.0–12.4] *μ*mol/L) than in arterial blood (2.7 [1.7–5.5] *μ*mol/L, *P *<* *0.0001) and the hepatic vein (3.4 [2.5–6.5] *μ*mol/L, *P *<* *0.0001) (Fig. 1A). Portal FGF19 levels (161 ± 78 pg/mL) were higher than arterial levels (135 ± 65 pg/mL, *P *=* *0.046), but not different from hepatic venous levels (146 ± 65 pg/mL, *P *=* *0.45) (Fig. 1B**)**. Portal bile salts were strongly related with bile salt levels in arterial (*ρ *= +0.80; *P *<* *0.001) and hepatic venous blood (*ρ *= +0.65; *P *<* *0.0001) (Fig. 2A). Likewise, portal venous FGF19 levels showed a strong correlation with arterial levels (*r* = +0.74, *P *<* *0.0001) and hepatic vein levels (*r* = +0.66, *P *<* *0.0001) (Fig. 2B). Transepithelial transport of bile salts is expected to induce ileal *FGF19* expression and subsequent release of FGF19 in the portal circulation. No correlation was observed between bile salts and FGF19 in portal venous blood under fasted conditions (*ρ* = +0.26, *P *=* *0.16) (Fig. 3A), whereas a positive association between FGF19 and bile salt levels was noted in arterial (*ρ* = +0.49, *P *=* *0.006) and hepatic venous blood (*ρ* = +0.42, *P *=* *0.02) (Fig. 3B & C).

### Visceral fluxes of bile salts and FGF19

**Figure 4:**
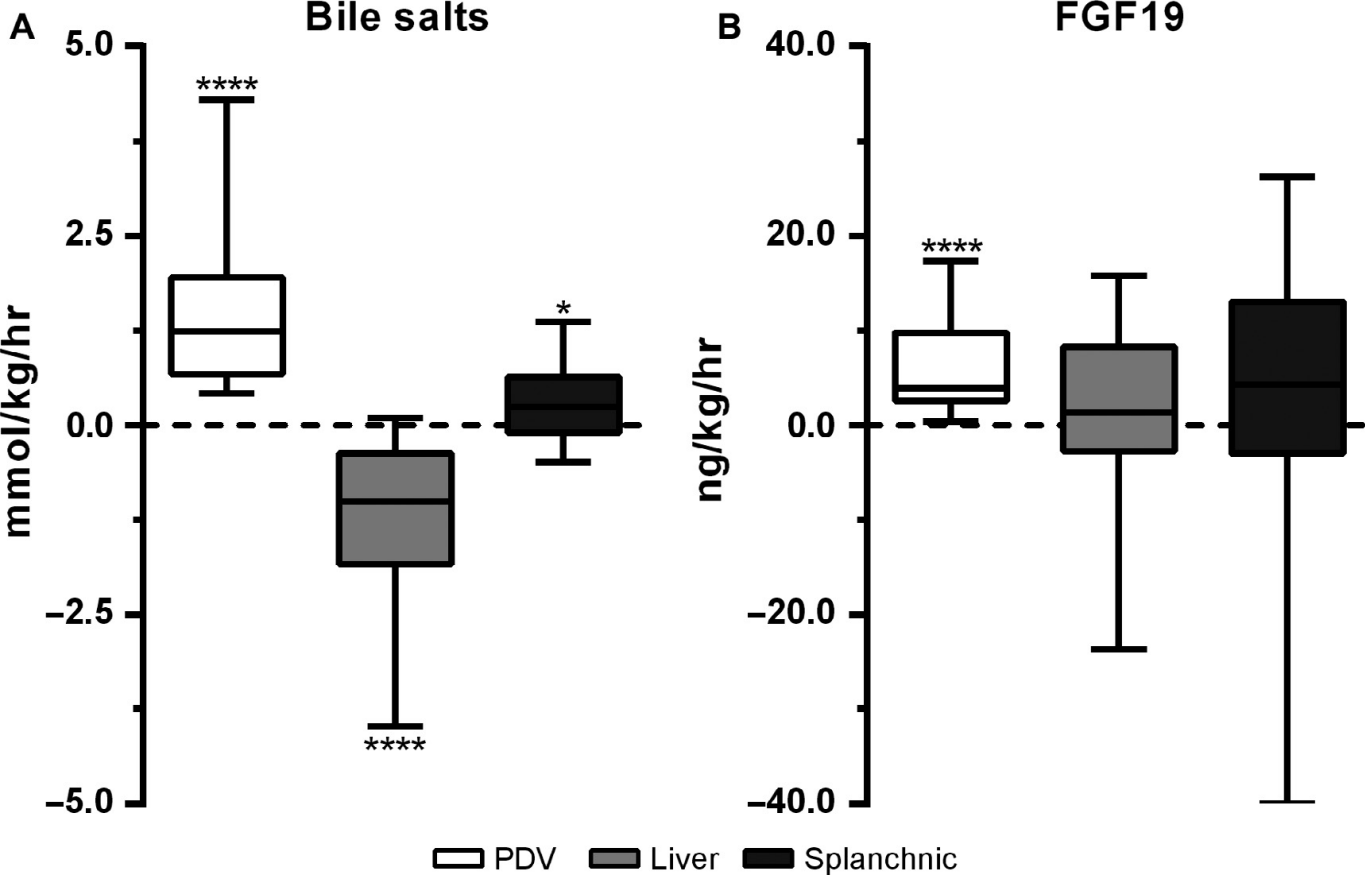
(A) Flux of FGF19 and (B) bile salts across the portal‐drained viscera (PDV), liver, and splanchnic area. Data are presented as box and whisker plots with 10th and 90th percentile. Positive values indicate net release and negative values indicate net uptake. *(*P *<* *0.05), **** (*P *<* *0.0001). FGF, Fibroblast growth factor.

The simultaneous sampling of blood from the vessels supplying and draining the viscera and the liver allows calculation of fluxes, thus providing insight into extraction or release of molecules by the PDV, the liver, and the splanchnic area. The median flux of bile salts across the PDV was positive (+1.2 [+0.7 to +2.0] mmol kg^−1^ h^−1^; *P *<* *0.0001) (Fig. 4A). Since bile salts are synthesized exclusively by the liver, the net release most likely reflects uptake and transepithelial transport of luminal bile salts. The presence of bile salts in the intestinal lumen in the fasted state is probably the consequence of the migratory motor complex that causes periodic partial contraction of the gallbladder in the interdigestive state.(Scott et al. 1983) The median flux across the liver was negative (−1.0 [−1.8 to −0.4] mmol kg^−1^ h^−1^; *P *<* *0.0001), indicating net uptake of bile salts from the portal blood. A minor spill‐over of bile salts into the systemic circulation was inferred from a positive splanchnic flux (+0.2 [−0.1 to +0.6] mmol kg^−1^ h^−1^; *P *=* *0.03).

In line with higher portal FGF19 levels, the flux of FGF19 across the PDV was positive (+4.0 [+2.1 to +9.9] ng kg^−1^ h^−1^, *P *<* *0.0001), indicating net release of FGF19 into the portal circulation (Fig. 4B). The flux of FGF19 across the liver (−0.2 [−3.7 to +7.4] ng kg^−1^ h^−1^; *P *=* *0.54.) was not significant, with a trend toward net release by the splanchnic organs (+3.6 [−7.2 to +11.8] ng kg^−1^ h^−1^; *P *=* *0.10.) (Fig. 4B).

## Discussion

In this study, we evaluated portal and systemic levels, as well as visceral fluxes of bile salts and FGF19 in human subjects under fasted conditions. Major findings of this study were elevation of FGF19 in the portal versus the systemic circulation, and net release of FGF19 by the portal‐drained viscera.

To our knowledge, this is the first demonstration that FGF19 is produced in vivo by visceral tissues draining in the portal circulation. What is the likely source of FGF19? Like its murine counterpart, human *FGF19* has a restricted pattern of expression. FGF19 is expressed in the ileum, with minor expression in other parts of the intestines (Zhang et al. 2013). Although *FGF19* is highly expressed in the human gallbladder and bile duct epithelium, expression at these sites is considered to result in release of FGF19 into bile, but not into the circulation (Zweers et al. 2012). Next‐generation sequencing efforts thus far failed to detect *FGF19* in pancreas, spleen, esophagus or stomach, thus, suggesting that net FGF19 release from the portal‐drained viscera is most likely derived from the ileum.

What could be the driving force for *FGF19* expression under fasted conditions? In rodents, intestine‐specific *Fxr* deficiency caused a two‐ to threefold decrease in ileal *Fgf15* expression (Kong et al. 2012). Bile duct ligation in mice, causing a complete absence of luminal bile salts, results in a 100‐fold decline of ileal *Fgf15* levels (Inagaki et al. 2006). Thus, these findings indicate that bile salts are responsible for regulating *FGF19/15* expression in the ileum. Although intestinal propulsive activity is likely reduced during anesthesia, the migratory motor complex results in a pulsatile delivery of bile salts to the intestinal lumen, and this may activate FXR and drive expression of *FGF19* under non‐fed conditions (Scott et al. 1983). The observed bile salt fluxes across the PDV, liver, and splanchnic area support this notion, and are consistent with uptake of bile salts from the intestinal lumen, release into the portal blood, and near‐complete reuptake by the liver.

Another interesting observation from this study was the strong correlation between portal and arterial FGF19 levels. Therefore, determining systemic levels can provide reliable information on portal FGF19 levels, at least under fasted conditions. Since bile salts regulate *FGF19* expression, a correlation between portal bile salts and FGF19 could be expected. Such relation was not apparent in this study set‐up, and this may relate to a lag time between *FGF19* gene transcription and protein translation/release.

Our data show that there is no significant flux of FGF19 across the liver. This indicates that hepatic clearance and production of FGF19 are absent, or at least in balance. Receptor‐mediated internalization of FGF ligands has been reported for members of the FGF family, but it is unclear if FGF19 may also be cleared in this way (Wiedlocha and Sorensen 2004). *FGF19* is not considered to be expressed in parenchymal cells of the healthy liver (Schaap et al. 2009), whereas potential *FGF19* expression in cholangiocytes is thought to result in biliary release (Zweers et al. 2012). Analysis of wedge resection specimens of patients of our study cohort, revealed a general lack of *FGF19* expression in grossly normal liver (data not shown).

Although no significant hepatic FGF19 flux was apparent in our study cohort, pathological conditions that prevent the enterohepatic cycling of bile salts are expected to affect FGF19 production by the small intestine and in certain conditions result in a shift toward hepatic production. For instance, failure of bile salts to enter the intestinal lumen in patients with malignant bile duct obstruction was accompanied by elevated FGF19 levels, likely due to adaptive expression of *FGF19* in the liver (Schaap et al. 2009). Thus, under cholestatic conditions a shift from intestinal toward hepatic FGF19 production seems to occur.

In summary, we demonstrate that under fasted conditions FGF19 is released by the PDV. Arterial levels of FGF19 strongly reflect portal venous levels of FGF19, and thus measurement of arterial levels can be used as indirect read‐out. Studies under stimulated conditions in humans are needed to gain further insights in the role of this signaling molecule in regulation of bile salt homeostasis.

## Conflict of Interest

None of the authors have anything to disclose regarding funding or conflict of interest with respect to this manuscript.
